# Elevated Levels of Plasma Phenylalanine in Schizophrenia: A Guanosine Triphosphate Cyclohydrolase-1 Metabolic Pathway Abnormality?

**DOI:** 10.1371/journal.pone.0085945

**Published:** 2014-01-21

**Authors:** Olaoluwa Okusaga, Olesja Muravitskaja, Dietmar Fuchs, Ayesha Ashraf, Sarah Hinman, Ina Giegling, Annette M. Hartmann, Bettina Konte, Marion Friedl, Jason Schiffman, Elliot Hong, Gloria Reeves, Maureen Groer, Robert Dantzer, Dan Rujescu, Teodor T. Postolache

**Affiliations:** 1 Mood and Anxiety Program, University of Maryland School of Medicine, Baltimore, Maryland, United States of America; 2 Department of Psychiatry and Behavioral Sciences, The University of Texas Health Science Center at Houston, Houston, Texas, United States of America; 3 Division of Biological Chemistry, Biocenter Innsbruck Medical University, Innsbruck, Austria; 4 Section of Molecular and Clinical Neurobiology, Ludwig Maximilians University, Munich, Germany; 5 Department of Psychology, University of Maryland, Baltimore, Maryland, United States of America; 6 Maryland Psychiatric Research Center (MPRC), Department of Psychiatry, University of Maryland School of Medicine, Baltimore, Maryland, United States of America; 7 Division of Child and Adolescent Psychiatry, University of Maryland School of Medicine, Baltimore, Maryland, United States of America; 8 University of Maryland Child and Adolescent Mental Health Innovations Center, Baltimore, Maryland, United States of America; 9 University of South Florida, Tampa, Florida, United States of America; 10 The University of Texas, MD Anderson Cancer Center, Houston, Texas, United States of America; 11 Department of Psychiatry, University of Halle-Wittenberg, Halle, Germany; 12 VISN 5 Capitol Health Care Network Mental Illness Research Education and Clinical Center (MIRECC), Baltimore, Maryland, United States of America; 13 VISN 19 MIRECC, Denver, Colorado, United States of America; The Nathan Kline Institute, United States of America

## Abstract

**Background:**

Phenylalanine and tyrosine are precursor amino acids required for the synthesis of dopamine, the main neurotransmitter implicated in the neurobiology of schizophrenia. Inflammation, increasingly implicated in schizophrenia, can impair the function of the enzyme Phenylalanine hydroxylase (PAH; which catalyzes the conversion of phenylalanine to tyrosine) and thus lead to elevated phenylalanine levels and reduced tyrosine levels. This study aimed to compare phenylalanine, tyrosine, and their ratio (a proxy for PAH function) in a relatively large sample of schizophrenia patients and healthy controls.

**Methods:**

We measured non-fasting plasma phenylalanine and tyrosine in 950 schizophrenia patients and 1000 healthy controls. We carried out multivariate analyses to compare log transformed phenylalanine, tyrosine, and phenylalanine:tyrosine ratio between patients and controls.

**Results:**

Compared to controls, schizophrenia patients had higher phenylalanine (p<0.0001) and phenylalanine: tyrosine ratio (p<0.0001) but tyrosine did not differ between the two groups (p = 0.596).

**Conclusions:**

Elevated phenylalanine and phenylalanine:tyrosine ratio in the blood of schizophrenia patients have to be replicated in longitudinal studies. The results may relate to an abnormal PAH function in schizophrenia that could become a target for novel preventative and interventional approaches.

## Introduction

Dopamine (DA) is a major neurotransmitter implicated in both the neurobiology of schizophrenia and the underlying mechanism of action of antipsychotic medications [1]. In the brain, phenylalanine hydroxylase (PAH) catalyzes the conversion of the essential amino acid phenylalanine (Phe) to tyrosine (Tyr). Tyr is then converted in a two-step enzymatic reaction (catalyzed sequentially by tyrosine hydroxylase (TH) and aromatic L-amino acid decarboxylase (L-AAAD)) to DA. Further downstream, two additional enzymes sequentially convert DA to norepinephrine (NE) and epinephrine (EP) respectively. Phe and Tyr both cross the blood-brain barrier [2] and the availability of these precursor amino acids may impact catecholamine synthesis in the brain [3]. Plasma Phe levels have been reported to be significantly elevated in schizophrenia patients relative to healthy controls [4], [5], [6], [7]; however, a few studies failed to find significant differences [8], [9], [10]. Plasma Tyr has been reported to be reduced in schizophrenia patients relative to healthy controls [7], [10]. DA and other catecholamine synthesis in the brain may be influenced not only by the availability of precursor amino acids, but also by the functional integrity of the various enzymes involved in the catecholamine synthetic pathway. In the synthesis of catecholamines, the committed step is the hydroxylation of Phe to Tyr by PAH; this step therefore requires strict regulation in order to ensure adequate homeostatic regulation of Phe [11]. The potential dire consequences of PAH dysfunction is exemplified by the metabolic disorder, phenylketonuria (PKU). PKU is characterized by hyperphenylalaninemia and, if untreated, can result in profound intellectual disability and neurologic sequelae [12]. Furthermore, the enzymes PAH, TH, and tryptophan-5-monoxygenase (TPH; which catalyzes the conversion of tryptophan to serotonin) require the cofactor 6R-L-erythro-5,6,7,8-tetrahydrobiopterin (BH4) for optimal function and BH4 deficiency can lead to hyperphenylalaninemia [13]. BH4 is synthesized *de novo* from guanosine triphosphate (GTP) which is initially converted to 7,8-dihydroneopterin triphosphate in a reaction catalyzed by GTP cyclohydrolase 1 (GTPCH-1); 6-pyruvoyltetrahydropterin synthase (PTPS) then catalyzes the conversion of 7,8-dihydroneopterin triphosphate to 6-pyruvoyltetrahydropterin; the third and final step is the conversion of 6-pyruvoyltetrahydropterin to BH4, a reaction catalyzed by sepiapterin reductase (SR) [14]. BH4 is ubiquitous and is likely present in all cells and tissues but current knowledge of the regulatory mechanism of BH4 in the brain is far from complete [14]. BH4 also modulates glutamatergic system function and regulates nitric oxide production [15]. BH4 deficit has previously been reported in schizophrenia patients relative to healthy controls [16].

Previous studies that have compared Phe and Tyr blood levels between schizophrenia patients and healthy controls have been relatively small with total sample sizes (i.e. cases and controls) less than 200 [6], [7], [10]. Some of the previous studies also did not explicitly indicate whether the diagnosis of schizophrenia was made by a structured clinical interview or by an unstructured evaluation by a psychiatrist [10]. In addition, the healthy controls in the previous studies were not thoroughly screened for mental illness. The aforementioned factors could have been responsible for some of the discrepancies in the results of the previous studies.

To overcome the limitations of small sample size, less precise methods of diagnosing schizophrenia in cases, and failure to thoroughly rule out the presence of mental illness in healthy controls, the present study compares plasma Phe, Tyr, and Phe:Tyr ratio in a large sample of schizophrenia patients diagnosed by the Structured Clinical Interview for DSM-IV (SCID) and adequately screened healthy controls. We hypothesized that Phe and Phe:Tyr ratio will be elevated in schizophrenia patients relative to controls and that Tyr level will be reduced in schizophrenia patients relative to controls.

## Materials and Methods

### Participants

The participants and the method of enrollment for this study have been previously described [17]. Briefly, we recruited 950 schizophrenia patients (ages 18 to 60 years, mean age 38.0±11.6 years) and 1000 healthy controls (ages 19 to 60 years, mean age 53.5±15.8 years) from the Munich area of Germany. All participants were white. The diagnosis of schizophrenia in all patients was confirmed with the SCID [18]. We used the SCID to confirm the absence of any lifetime psychotic disorder in the healthy control group. We excluded individuals with schizoaffective disorder, schizophreniform disorder, substance-induced psychosis, and psychotic disorder NOS. Symptom severity in all patients was measured using the Positive and Negative Syndrome Scale (PANSS) [19]. All patients were treated with antipsychotic medications, the doses of which were expressed in chlorpromazine equivalents [20]. Blood samples were obtained without any dietary or fasting protocols. Participants were recruited from inpatient and outpatient settings.

### Ethics Statement

The study procedures were described in detail to all participants, after which written informed consent was obtained. The majority of included patients had full capacity to consent. The ability to consent was determined by a psychiatrist. In exceptional cases where a patient’s capacity to consent was in doubt, but the patient and his/her legal guardian wished to participate, written informed consent was obtained from both the patient and the legal guardian. The local ethics committee of Ludwig Maximilians University, Munich, Germany approved the study and it was determined as exempt by the Institutional Review Board of the University of Maryland School of Medicine Baltimore, MD, USA.

### Plasma Measurement of Phe and Tyr

Blood was obtained via a forearm vein and drawn in EDTA containing tubes. No fasting protocol was utilized. The samples were centrifuged for 10 min at 4°C and the resulting plasma aliquoted into Eppendorf tubes which were frozen immediately at −80°C. Samples were kept frozen at −80°C until analysis. Phe and Tyr concentrations were determined by high performance liquid chromatography (HPLC) monitoring their natural fluorescence at an excitation wavelength of 210 nm and an emission wavelength of 302 nm as described by Neurauter et al [21]. 100 µL plasma was diluted with 100 µL of 500 µM 3-nitro-L-tyrosine (internal standard) and 25 µL of 2 M trichloroacetic acid was used to precipitate and separate proteins. Supernatants of the samples were diluted 1∶25 with 0.015 M potassium dihydrogen-phosphate after centrifugation and the supernatant was used as elution buffer on HPLC. An albumin-based calibration mixture was prepared in parallel to the sera, and it contained 100 µM Phe and 100 µM Tyr and underwent the same pre-analytical procedures as plasma specimens.

### Statistical Analyses

To estimate the activity of PAH, the ratio of the substrate Phe versus the concentrations of the enzyme product Tyr (Phe:Tyr ratio = Phe/Tyr) was calculated [22]. The distributions of Phe, Tyr, and Phe:Tyr ratio were skewed to the right and we therefore applied a logarithmic transformation to normalize these variables. We checked for normality by evaluating histogram plots, skewness, and kurtosis. We also computed categorical variables by initially splitting the levels of Phe, Tyr, and Phe:Tyr ratio into four quartiles after which we combined the lower three quartiles for Phe and Phe:Tyr ratio and the upper three quartiles for Tyr. The resulting binary categories contained the upper 25th percentile and lower 75th percentile for Phe and Phe:Tyr ratio and the upper 75th percentile and lower 25th percentile for Tyr. We used t-tests and χ2 test to compare continuous and categorical demographic variables between schizophrenia patients and healthy controls. T-tests were used for unadjusted comparison of log-transformed Phe, Phe:Tyr ratio, and Tyr between patients and controls and linear regression analyses were used to compare groups adjusted for gender, age, education (a proxy for socio-economic status), and body mass index (BMI). We have presented geometric means with 95% confidence intervals obtained by exponentiating mean log-transformed Phe, Phe:Tyr ratio, and Tyr for adjusted and unadjusted comparisons of the two groups. Post-hoc, we evaluated the relationship between having a diagnosis of schizophrenia and Phe and Phe:Tyr ratio in the upper 25th percentile or Tyr in the lower 25th percentile using χ2 test. We calculated odds ratio associated with Phe and Phe:Tyr ratio in the upper 25th percentile or Tyr in the lower 25th percentile in schizophrenia patients using logistic regression adjusted for gender, age, education, and BMI. All significance levels reported are two-sided with P values <0.01 considered statistically significant. We carried out all the statistical analyses using IBM SPSS version 20 (Armonk, NY: IBM Corp).

## Results

### Sample Characteristics

As shown in table 1, the schizophrenia patients in this sample were on average 15 years younger, had higher BMI, and less education compared to the healthy controls. There were more males among patients than among controls. The mean PANSS score of 101.5 means that the average patient in this sample can be classified as “markedly ill” based on the classification by Leucht et al [23].

**Table 1:** Demographic and clinical characteristics of the study groups (n = 1, 950).

Characteristics	Healthy Controls(n = 1000)	Schizophrenia Patients(n = 950)	p-value*
Age, years (mean ± SD)	53.5±15.8	38.0±11.6	<0.0001
BMI (mean ± SD)	24.8±3.9	27.1±5.5	<0.0001
Gender male, n (%)	490 (49.0)	600 (63.2)	<0.0001
Education Level (n, %)			<0.0001
Primary	246 (24.7)	405 (42.7)	
Secondary	302 (30.2)	241 (25.4)	
Tertiary	451 (45.1)	303 (31.9)	
Duration of illness, months (mean ± SD)	–	13.1±16.2	
Mean dose of antipsychotic in CPZ equivalent(mean ± SD)		475±1248	
PANSS (mean ± SD)			
Positive symptoms	–	27.7±6.4	
Negative symptoms	–	24.4±7.5	
General	–	49.4±11.7	
Total score	–	101.5±21.4	

* *x* ^2^ test for categorical variables, t-test for continuous variables.

BMI = body mass index; CPZ = chlorpromazine.

### Phe, Tyr, and Phe:Tyr Ratio between Groups

Schizophrenia patients had significantly higher Phe (geometric mean difference 1.26 µmol/L; CI 1.18 to 1.36, p<0.0001) and Phe:Tyr ratio (geometric mean difference 1.41; CI 1.33 to 1.48, p<0.0001) compared to healthy controls and this finding persisted after controlling for gender, age, education, and BMI differences between the 2 groups (table 2). Tyr levels did not significantly differ between patients and controls in unadjusted and adjusted analysis (p = 0.596, p = 0.668 respectively).

**Table 2:** Unadjusted and adjusted geometric mean differences in Phenylalanine, Tyrosine and Phenylalanine:Tyrosine ratio for schizophrenia patients vs. healthy controls (reference group is the healthy controls).

	Geometric mean^a^ differences	95% CI	p-value
Unadjusted			
Phenylalanine	1.26 µmol/L	1.18 to 1.36	<0.0001
Tyrosine	0.98 µmol/L	0.91 to 1.05	0.596
Phenylalanine:Tyrosine ratio	1.41	1.33 to 1.48	<0.0001
Adjusted^b^			
Phenylalanine	1.19 µmol/L	1.08 to 1.30	<0.0001
Tyrosine	0.98 µmol/L	0.91 to 1.07	0.668
Phenylalanine:Tyrosine ratio	1.30	1.22 to 1.39	<0.0001

a Based on back-transformed means of log transformed values.

b Adjusted for gender, age, education and body mass index (BMI).

### Relationship between having a Diagnosis of Schizophrenia and Percentile Category of Phe, Tyr, and Phe:Tyr Ratio

From χ2 analysis, having a diagnosis of schizophrenia was associated with Phe level (χ2 23.61, p<0.0001) and Phe:Tyr ratio (χ2 199.75, p<0.0001) in the upper 25th percentile and Tyr levels in the lower 25th percentile (χ2 30.33, p<0.0001). There was an increased likelihood of having Phe levels (OR 1.87; CI 1.43 to 2.43, p<0.0001) and Phe:Tyr ratio (OR 4.27; CI 3.22 to 5.67, p<0.0001) in the upper 25th percentile in schizophrenia patients relative to controls. The likelihood of having Tyr levels in the lower 25th percentile was increased in schizophrenia patients (OR 1.86; CI 1.43 to 2.43, p<0.0001).

## Discussion

In this relatively large sample, schizophrenia patients had significantly higher plasma Phe and Phe:Tyr ratio compared to healthy controls. Tyr levels did not differ between the two groups; however, having a diagnosis of schizophrenia was associated with Tyr in the lower 25^th^ percentile with increased odds of Tyr in the associated percentile. Our finding of elevated plasma Phe in schizophrenia is consistent with the results of two previous smaller studies [6], [7]. However, the absence of any statistically significant difference in plasma Tyr between patients and controls in the current study differs from the reports by Wei et al [10] and Rao et al [7], both of which found reduced plasma Tyr levels in schizophrenia patients.

The relatively raised levels of Phe in schizophrenia patients could be a result of abnormal metabolism of the essential amino acid. For example, abnormal Phe kinetics was recently documented in schizophrenia patients relative to healthy controls via the measurement of radioactive carbon (^13^CO2) in the breath [^13^C-phenylalanine breath test (^13^C-PBT)] of the subjects after the oral administration of ^13^C-phenylalanine [24]. Elevated levels of Phe:Tyr ratio have been associated with immune activation and inflammation in individuals suffering from cancer, infection, and trauma [21], [25], [26] and a recent study also found Phe:Tyr ratio to be increased in patients infected with hepatitis C virus treated with the immune modulator interferon-α [27]. Furthermore, immune activation with increased circulating levels of proinflammatory cytokines have also been consistently reported in schizophrenia patients [28]. Though speculative, as we did not measure markers of immune activation such as cytokines, C-reactive protein, or neopterin, it is possible that an immune activated state in the schizophrenia patients contributes to the inhibition of PAH and this in turn could be responsible for the elevated Phe and Phe:Tyr ratio. Oxidative stress accompanying immune activation of macrophages (via release of reactive oxygen species and neopterin) has been demonstrated as the chemical background for the deactivation of PAH [11], a finding further supported by a study which found Phe levels to be correlated with the marker of oxidative stress, isoprostane-8 [21].

PAH also requires the cofactor BH4 for optimal activity and inflammatory cytokines such as interferon-γ (the levels of which have been reported to be elevated in schizophrenia [28]) stimulate GTPCH-1, the first enzyme involved in the synthesis of BH4; however, due to the relative deficiency of an intermediate enzyme, 6-pyruvoyltetrahydropterin synthase, there is reduced BH4 synthesis in immune-activated states [29]. The BH4 synthesized by other cells can also be degraded by the reactive oxygen species produced by immune cells [30], [31]. Therefore, the elevated Phe and Phe:Tyr observed in schizophrenia patients in the current study could also be related to a quantitative and qualitative deficiency of BH4. A study by Richardson and colleagues [16] which found reduced plasma BH4 in schizophrenia patients lends support to this hypothesis.

It is important to note that even though Phe levels are elevated in schizophrenia patients, the elevated levels observed in our sample (as well as those by Rao et al [7] and Bjerkenstedt et al [6]) are not of the same severity as in PKU. To illustrate, the patient with the highest plasma Phe in our sample had a value of 266 µmol/L, a value in the benign hyperphenylalaninemia phentotypic classification of PKU [32]. Patients with benign hyperphenylalaninemia may require Phe level monitoring, but do not need any special dietary modification as is necessary for individuals with classical PKU [30]. Normal plasma Phe ranges from 30 to 80 µmol/L [33].

### Limitations

Antipsychotic medications (taken by all participants with schizophrenia and none of the controls) are potential confounders, as it is conceivable that they may influence levels of Phe and Tyr. Previous research, however, suggests that increased plasma Phe in schizophrenia patients is unlikely due to antipsychotic medication as all the patients in the study by Bjerkenstedt et al [6] were unmedicated and both medicated and unmedicated patients had elevated plasma Phe in the study by Rao et al [7]. The cross sectional design of the study was also a limitation, limiting the ability to ascribe causality or a direction of effect between illness and the measured amino acids. Additionally, it would have been highly informative if we had been able to relate the elevated Phe:Tyr ratio (a proxy for PAH and GTPCH-1 pathway function) directly with inflammation, but the unavailability of enough plasma to assay inflammatory markers (e.g. interleukin-6, highly sensitive C-reactive protein, or neopterin) was prohibitive. We were also unable to measure Phe and Tyr centrally (cerebrospinal fluid- CSF). Although patients differed considerably from controls on demographic variables, statistical analyses controlled for these factors.

Another limitation is not using a fasting protocol or gathering information from food diaries which are potential sources of bias since food preferences, access, as well as the timing of meals could differ between medicated patients with schizophrenia and psychiatrically healthy individuals. Temporal proximity to meals and the amount of protein/amino acids in the diet do influence Phe and Tyr levels in humans [34] but a diurnal variation in amino acid levels also exist and is likely multifactorial [34], [35]. With regard to the acute effects of amino acid ingestion on plasma levels, Leeming et al [36] found that the peak value of plasma Phe occurred one hour after an oral load of Phe while Tyr peak plasma levels were observed between two to two and a half hours after an oral load.

Despite these limitations, the study included several strengths such as a relatively large sample size, confirmation of schizophrenia diagnosis by SCID, exclusion of mental illness (including personality disorders) in healthy controls, and a low diagnostic heterogeneity by including only schizophrenia patients. While acknowledging the limitation of not measuring the amino acids in the CSF, there is also an advantage to measuring in the blood as it is less invasive and more practical to monitor blood in patients rather than the CSF.

In summary we have reported that Phe and Phe:Tyr ratio are elevated in schizophrenia patients relative to healthy controls, and that lower levels of Tyr are more common among schizophrenia patients, findings which we have hypothesized to be likely related to immune activation and impaired PAH function. Longitudinal studies with improved methodology (fasting blood levels, accounting for diet using food diaries, and measuring markers of inflammation) are needed to evaluate the effect of changes in plasma Phe on psychopathology in schizophrenia patients. Randomized controlled trials of Phe-lowering interventions in schizophrenia will also contribute to a better understanding of the effect of Phe homeostasis on schizophrenia and could lead to the development of novel preventative and treatment strategies for this highly prevalent and severe mental illness.
